# The Role of Migration in Maintaining the Transmission of Avian Influenza in Waterfowl: A Multisite Multispecies Transmission Model along East Asian-Australian Flyway

**DOI:** 10.1155/2018/3420535

**Published:** 2018-03-26

**Authors:** Akira Endo, Hiroshi Nishiura

**Affiliations:** Graduate School of Medicine, Hokkaido University, Kita 15 Jo Nishi 7 Chome, Kitaku, Sapporo 0608638, Japan

## Abstract

**Background:**

Migratory waterfowl annually migrate over the continents along the routes known as flyways, serving as carriers of avian influenza virus across distant locations. Prevalence of influenza varies with species, and there are also geographical and temporal variations. However, the role of long-distance migration in multispecies transmission dynamics has yet to be understood. We constructed a mathematical model to capture the global dynamics of avian influenza, identifying species and locations that contribute to sustaining transmission.

**Methods:**

We devised a multisite, multispecies SIS (susceptible-infectious-susceptible) model, and estimated transmission rates within and between species in each geographical location from prevalence data. Parameters were directly sampled from posterior distribution under Bayesian inference framework. We then analyzed contribution of each species in each location to the global patterns of influenza transmission.

**Results:**

Transmission and migration parameters were estimated by Bayesian posterior sampling. The basic reproduction number was estimated at 1.1, slightly above the endemic threshold. Mallard was found to be the most important host with the highest transmission potential, and high- and middle-latitude regions appeared to act as hotspots of influenza transmission. The local reproduction number suggested that the prevalence of avian influenza in the Oceania region is dependent on the inflow of infected birds from other regions.

**Conclusion:**

Mallard exhibited the highest transmission rate among the species explored. Migration was suggested to be a key factor of the global prevalence of avian influenza, as transmission is locally sustainable only in the northern hemisphere, and the virus could be extinct in the Oceania region without migration.

## 1. Introduction

Migratory waterfowl are deemed as important host of maintaining avian influenza. Waterfowl annually migrate over the continents along the routes known as migratory flyways, serving as carriers of virus across distant sites [1–4]. Published studies suggested that the prevalence of influenza virus varies among different bird species (most frequently isolated from dabbling ducks including mallards) and that there are also geographical and temporal variations [5–7].

Such variations in the frequency of influenza virus may result from heterogeneous nature of the transmission dynamics including susceptibility, climate effect, and population dynamics along with the ecological behavior of these waterbirds such as stopover, feeding, and breeding. The long-distance migration of the waterbirds is thus expected to play an important role in transmission, but the relevance of multispecies transmission dynamics to the global patterns of avian influenza have yet to be explored.

The majority of avian influenza strains are believed to be scarcely transmissible in the human population, and thus, usually confined to bird species. However, sporadic spillover events have been frequently observed in the last few decades [8–10]. Previous studies demonstrated the certain infectiousness to humans [11] and also the substantial potential of human-to-human transmissibility acquired by spontaneous mutations or via reassortment with human (or swine) influenza viruses [12, 13]. The emergence of such novel virus with higher transmission potential could lead to a serious worldwide pandemic, and thus, clarifying the natural history, ecological behavior of the hosts, and any indication of ongoing genetic changes would be of utmost practical importance.

A published study modelled interspecies transmission dynamics of avian influenza by employing the so-called SIS (susceptible-infectious-susceptible) model, offering the definition of the reservoir species using the eigenvalue of the projected next generation matrix [14]. Mallard and dabbling ducks were identified as important hosts of influenza A virus from the quantified next generation matrix based on species-specific prevalence data. Adopting a similar approach, the present study further incorporates geographical variations and the migration between different sites into the model. Applying this multisite, multispecies transmission model to the existing field sample data in the regions along East Asian-Australian Flyway (EAAF), the present study aims to capture the global dynamics of avian influenza and to identify species and geographical locations that essentially contribute to sustaining transmission.

## 2. Method

### 2.1. Data Source

We investigated the multispecies avian influenza prevalence data in countries that belong to EAAF (USSR, Japan, Australia, and New Zealand) from Olsen et al. and De Marco et al. [5, 15], retrieving the sample dataset from vent (anus) swab or fresh droppings. We focused on four genera (*Anas*, *Cygnus*, *Larus*, and *Sterna*), which have been intensively surveyed for influenza in the EAAF regions and are ecologically important for virus circulation. As mallard (*Anas platyrhynchos*) has been considered as the reservoir species [5, 14], we classified the waterfowl into three distinct groups: mallard, ducks (*Anas* species except for mallard), and other species (*Cygnus*, *Larus*, and *Sterna*). Samples from selected countries were assumed to represent one of the three discrete geographic regions: high-latitude area (>50° N), mid-latitude area (<50° N), and Oceania (Australia and New Zealand). The number of positive/negative samples was counted for each group and area.

### 2.2. Model

Let *i*_*kg*_(*t*) be the prevalence in species *k* in site *g*. The multisite multispecies SIS model is described by a set of ordinary differential equations (ODE):

(1) $$\begin{array}{c} \frac{d}{dt}i_{kg}\left(t\right)=\left(1-i_{kg}\left(t\right)\right)\sum_{l}\beta_{kl,g}i_{lg}\left(t\right)-\left(\gamma_{k}+\mu_{k}\right)i_{kg}\left(t\right)+\sum_{h}\frac{m_{k,gh}i_{kh}N_{kh}}{N_{kg}}, \end{array}$$

where *β*_*kl*,*g*_ is interspecies transmission rate from species *l* to *k* and *m*_*k*,*gh*_ is migration rate from site *h* to *g* (*m*_*k*,*gg*_=−∑_*h*_ *m*_*k*,*hg*_). *N*_*kg*_ is the population size of species *k* at site *g*. *γ*_*k*_ and *μ*_*k*_ are recovery and mortality (birth) rate, respectively, of species *k*.

**Figure 1:**
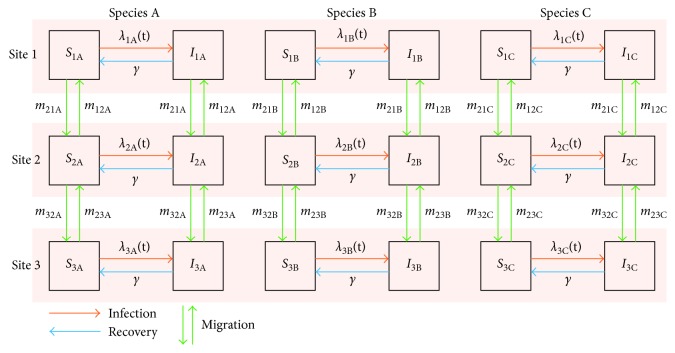
A schematic diagram of the multisite multispecies compartment model. Susceptible-Infectious-Susceptible (SIS) model was used, and population of each species at each site was classified as either susceptible or infectious; for example, *S*_1A_ (*I*_1A_) represents the susceptible (infectious) compartment of species A at site 1. Variables beside the arrows show the rate of transition from one compartment to another (different notations from the main text may be used for generality). Pink-shaded areas indicate the mixing of hosts, reflecting our assumption that mixing can be cross-species but not cross-site. The force of infection *λ*(*t*) is thus dependent on all the infectious compartments at the same site.

In the present study, we consider 3 species and 3 locations (schematic diagram shown in Figure 1). Assuming that direct migration between locations 1 and 3 (i.e., between high-latitude and Oceania area) is negligible and that population distribution is at the equilibrium, we get

(2) $$\begin{array}{c} \begin{array}{l} m_{k,13}=m_{k,31}=0,\\ m_{k,gh}N_{kh}=m_{k,hg}N_{kg}. \end{array} \end{array}$$

Equilibrium is determined by the matrix form equation

(3) $$\begin{array}{c} \mathrm{diag}\left(1-i_{\mathrm{Eq}}\right)\left(\begin{array}{ccc} B_{1} & O & O\\ O & B_{2} & O\\ O & O & B_{3} \end{array}\right)i_{\mathrm{Eq}}-\mathrm{diag}\left(\gamma +\mu \right)i_{\mathrm{Eq}}+\mathrm{diag}{\left(N\right)}^{-1}\left(\begin{array}{ccc} -M_{12} & M_{12} & O\\ M_{12} & -M_{12}-M_{23} & M_{23}\\ O & M_{23} & M_{23} \end{array}\right)i_{\text{Eq}}=0. \end{array}$$

To let the equations be solvable, *M*_12_ and *M*_23_ were assumed to be proportional. We parameterized the species-specific migration rate as $M_{0}=\left(\begin{array}{ccc} m_{1} & 0 & 0\\ 0 & m_{2} & 0\\ 0 & 0 & m_{3} \end{array}\right)$ and expressed the migration rates by employing a logistic function logis(*r*)=(1+exp(−*r*))^−1^, that is, *M*_12_=logis(−*r*)*M*_0_ and *M*_23_=logis(*r*)*M*_0_.

We assumed that the transmission matrix in each site is characterized by the site-specific coefficient *β*_*g*_ and Newman's assortativity parameter *θ*:

(4) $$\begin{array}{c} B_{g}=\beta_{g}\left(\begin{array}{ccc} c_{1}^{2}n_{1g}\left(1-\theta \right)+\theta c_{1}^{2} & c_{1}c_{2}n_{1g}\left(0\right) & c_{1}c_{3}n_{1g}\left(1-\theta \right)\\ c_{1}c_{2}n_{2g}\left(1-\theta \right) & c_{2}^{2}n_{2g}\left(1-\theta \right)+\theta c_{2}^{2} & c_{2}c_{3}n_{2g}\left(1-\theta \right)\\ c_{1}c_{3}n_{3g}\left(1-\theta \right) & c_{2}c_{3}n_{3g}\left(1-\theta \right) & c_{3}^{2}n_{3g}\left(1-\theta \right)+\theta c_{3}^{2} \end{array}\right),\;\left(g=1,\;\;2,\;\;3\right), \end{array}$$

where *n*_*kg*_=*N*_*kg*_/∑_*k*_ *N*_*kg*_.

The basic reproduction number was derived as the largest eigenvalue of the next generation matrix

(5) $$\begin{array}{c} N.G.M.=B\Sigma^{-1}, \end{array}$$

where

(6) $$\begin{array}{c} \begin{array}{l} B=\left(\begin{array}{ccc} B_{1} & O & O\\ O & B_{2} & O\\ O & O & B_{3} \end{array}\right),\\ \Sigma =\mathrm{diag}\left(\gamma +\mu \right)-\left(\begin{array}{ccc} -M_{12} & M_{12} & O\\ M_{12} & -M_{12}-M_{23} & M_{23}\\ O & M_{23} & -M_{23} \end{array}\right). \end{array} \end{array}$$

To quantify the role of each species in local transmission, we extracted the local components of the next generation matrix where migration term is removed:

(7) $$\begin{array}{c} R_{g}=B_{g}{\left(\mathrm{diag}\left(\gamma +\mu \right)\right)}^{-1}, \end{array}$$

and defined the species-specific local reproduction number as the largest eigenvalue of *R*_*g*_(*K*), the submatrix of *R*_*g*_ corresponding to a set of species *K*. The (overall) local reproduction number is the largest eigenvalue of *R*_*g*_, corresponding to *K*={Mallard, Ducks, Others}.

### 2.3. Parameter Settings and Posterior Sampling

The recovery rate *γ* was borrowed from the literature (1/11.09 (day^−1^); a reciprocal of the infectious period for low pathogenic avian influenza. The average of estimates for adult and young birds was used) [16], and was assumed to be identical regardless of species and location. Mortality rate *μ* was considered as negligible compared with *γ*, as the life expectancy of waterfowl species studied ranged from a few years to decades. Mallard accounted for approximately 20% of the breeding population of ducks in the USA [17], and thus we assumed that the relative population sizes of mallard, ducks, and others are 1:4:5. Estimates of regional population distribution and migration rate were scarcely available; we adopted a rough assumption that the relative population distribution is 1:1:1 in the three areas, and that the average migration rate over the three species groups is 50% of the maximum mobility (i.e., the situation where all waterfowl fly around the three areas annually). The sensitivity of the results was tested against the variation in these assumptions.

Employing the non-informative prior, the posterior distribution for each parameter was sampled by solving (3), where prevalence *i*_Eq_ was drawn from the beta distribution. Equation was solved by minimizing the squared relative error (SRE):

(8) $$\begin{array}{c} \text{SRE}=\sum_{j}{\left(\frac{E_{j}}{i_{j}}\right)}^{2}, \end{array}$$

where *i*_*j*_ and *E*_*j*_ are the *j*th components of *i*_Eq_ and the left-hand side of (3), respectively. Samples were discarded if their SRE exceeded 0.02 (which corresponds to approximately 5% error for each component on average), as the majority of such samples yielded unrealistic parameter values (e.g., extremely small transmission rate).

## 3. Results

**Figure 2:**
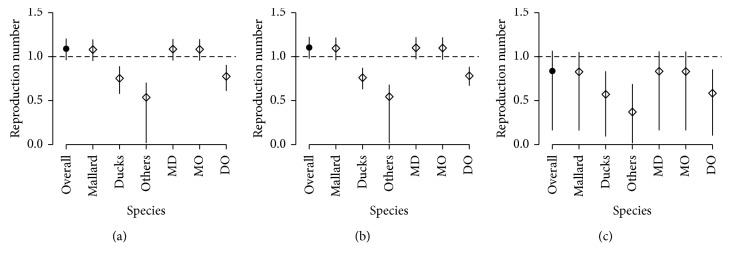
The species-specific local reproduction numbers of avian influenza estimated from prevalence data. (a) High-latitude area, (b) mid-latitude area, and (c) Oceania. The dots represent median and whiskers the 95% credible intervals. MD, MO, and DO refer to the pairs of species, {Mallard, Ducks}, {Mallard, Others}, and {Ducks, Others}, respectively. Gray dotted line denotes the threshold of 1.

In the high-latitude region, prevalence of mallard, ducks, and others were 4/61, 38/1595, and 8/140. Similarly, the prevalence in mid-latitude were 35/516, 89/3319, and 91/4461, while those in Oceania were 8/383, 15/348, and 1/419, respectively. Parameters were estimated by posterior sampling, and the next generation matrix was derived from the samples (Tables 1 and 2). The basic reproduction number was estimated at 1.1 (95% credible intervals (CrI): 1.0–1.2), significantly above the value of 1 reflecting the sustained transmission of the virus in the population. High- and mid-latitude areas were found to be the most frequent sites of transmission, and mallard had the highest transmissibility. Migration rate of “Others” which includes swans, gulls, and terns were more than 10 times higher than those of mallard and ducks, reflecting the exceedingly long-distance migration routes taken by those species [18–20]. The species-specific local reproduction numbers displayed in Figure 2 indicated that high-latitude and mid-latitude areas play a critical role in sustaining transmission, and that mallard is the major driver of the endemic. On the other hand, the overall (or any other species-specific) local reproduction number in Oceania areas was below 1, suggesting that the prevalence of avian influenza in this area is dependent on the inflow of infectious birds from the other areas.

**Figure 3:**
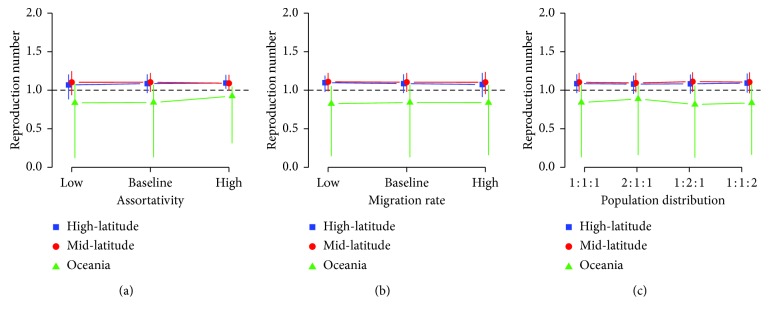
Sensitivity analysis against assortativity, average migration rate, and population distribution. The local reproduction numbers in each geographical area are compared. (a) The local reproduction numbers for different assortativity: 0.7 (low), 0.8 (baseline), and 0.9 (high); (b) average migration rate: 25% (low), 50% (baseline), and 100% (high) of the maximum mobility (i.e., all birds migrate around the three areas annually); (c) population distribution: high-latitude:mid-latitude:Oceania = 1:1:1, 2:1:1, 1:2:1, and 1:1:2.

We conducted a sensitivity analysis by varying assortativity, migration rate, and population distribution, which we set based on a rough assumption without being backed up by empirical data (Figure 3). Given that our baseline values being assortativity at 0.8, migration rate at 50% (i.e., the total sum of migration flow rates between locations is 50% of the total population per year), and population distributed 1:1:1 in three areas, we varied each of these and compared the estimated local reproduction number. Overall, the changes in these assumptions did not qualitatively change the outcome, while in some settings we observed slight variations. High assortativity led to the elevation of the estimated local reproduction number in the Oceania area. The reproduction number in Oceania also seemed affected by the population size in the high-latitude area. However, those changes were subtle and qualitatively negligible.

## 4. Discussion

The present study applied the multisite, multispecies SIS model to the field sample data of influenza virus from migratory waterfowl. Parameter estimates suggested that the northern hemisphere is the hotspot of avian influenza transmission, and that mallard play the most significant role in the circulation of the virus. The migration rate of the species other than ducks (i.e., *Cygnus*, *Larus*, and *Sterna*) were far greater than that of ducks reflecting the long migration distance of those species (from several thousands to tens of thousands kilometers) [18–20].

Migration of waterfowl has been considered as an important factor for characterizing the global distribution of avian influenza. The estimated local reproduction numbers suggested that migratory waterfowl carry influenza virus along EAAF and cause continuous transmission in the Southern Hemisphere, where the reproduction of avian influenza is not locally sustainable. The local reproduction number in the Oceania area was 0.8, indicating that the virus transmission cannot be sustained only by the local transmission, and that the circulation of the virus is likely to be dependent on the incoming infected waterfowl.

It is believed that avian influenza is frequently transmitted among migratory waterfowl in the arctic breeding sites and in the slightly southern area where they concentrate before the long-distance migration [7, 21]. Our findings also confirmed the importance of northern part of the flyways in the avian influenza transmission, with the local reproduction number above 1 for the two regions in the Northern Hemisphere. The role of the pan-Arctic region is all the more emphasized by the possibility that avian influenza virus might survive the winter period in water and ice and be transmitted via the contaminated water in the next year [22]. Although this possible overwintering transmission via water source was not captured by our model, it might even increase the potential of sustained transmission of avian influenza in the Arctic region as the persistence of the virus can be almost year-long at lower temperature [23, 24].

Although migratory birds play critical roles in carrying avian influenza viruses from one region to another, it is unlikely that disease control interventions can effectively target wild bird population. Rather, the effort may be put on monitoring the virus prevalence and emergence, and then on interventions focusing on the surface of potential spillover (e.g., poultry market) once the risk becomes apparent. We believe that our study highlighted the importance of continuous surveillance of migratory birds, at least in the Northern Hemisphere regions. Besides, as it was indicated that the virus prevalence in Oceania regions is dependent on the migration from the north, surveillance for avian influenza in Oceania countries could be intensified in response to the results in the northern EAAF countries, where a novel strain emerged is likely to be amplified in the wild bird population before it is brought in via migration. When more abundant data are accumulated through high-quality surveillance and further advances are made in studies on eco-evolutionary dynamics of avian influenza viruses, we might even be able to develop a predictive model for the geospatial spread of mutant strains; our model and its implications would provide insightful clues to the basic concepts and strategies for such attempts in the future.

Our study holds multiple limitations. First, we did not have an access to the temporal data of influenza prevalence and migration patterns of the waterbirds. Currently, collecting biological samples from birds is the only feasible method for estimating disease prevalence in wild birds, and new insights into the incidence estimation are called for. The available datasets were not sufficient for temporal analysis, and thus we limited our analysis to the equilibrium state, as have been the case for most of the previous wildlife models. As migratory birds are in different locations depending on the season, seasonal variation in prevalence reported in the previous study [5] might be driven by immunological, seasonal, or geographical factors. Such complex temporal dynamics was neglected in the present study and remains for the future work. Second, geographical distribution and migration patterns of waterfowl were radically simplified. Migration patterns of wild birds have been a keen focus of scientists, and the technology has advanced to track the route of migratory birds, and many species have been under observation [25–27]. However, the whole picture of migratory birds is yet to be thoroughly understood. Recent studies utilize Global Positioning System to directly track the migration routes of wild birds, but such attempts are confronted by the limited power source the birds can carry [28]. The cost of the device is another obstacle that limits the sample size. Instead of accounting for the detailed geographical properties of migration behavior, we simplified the migration patterns of waterfowl into a multisite ODE model in the present study. Third, strong assumptions on the model structure were made to reduce the number of parameters. We believe that our model was not unreasonably structured, but it should be noted that our results may only reflect overall characteristics and the possible interaction between variables (e.g., regional variance in the interspecies mixing rate) could have been smoothed out.

While our exercise suffers from these limitations, we believe that our simple multisite, multispecies transmission model successfully captured the global patterns of avian influenza prevalence, reflecting the effect of migration along the flyway. As the reproduction of the virus was suggested to be sustainable only in the Northern Hemisphere, efforts to clarify its natural history along with the host behavior in these regions would be of greater importance to understand the disease dynamics and to better prepare for the possible spillover of highly pathogenic avian influenza.

## Figures and Tables

**Table 1:** Parameter estimates of the multisites multispecies transmission model.

Parameter	Notation	Estimate (95% CrI)
Regional coefficient	*β*_1_ (high-latitude)	1.082 (0.946, 1.394)
	*β*_2_ (mid-latitude)	1.102 (0.963, 1.394)
	*β*_3_ (Oceania)	0.823 (0.201, 1.036)
Contact rate	*c*_1_ (mallard)	0.331 (0.296, 0.338)
	*c*_2_ (ducks)	0.266 (0.223, 0.294)
	*c*_3_ (others)	0.219 (0.040, 0.262)
Species-specific migration rate	*m*_1_ (mallard)	0.00281 (0.00052, 0.01127)
	*m*_2_ (ducks)	0.00356 (0.00066, 0.01414)
	*m*_3_ (others)	0.02648 (0.00759, 0.03170)
Logarithm of the ratio of migration between areas	*r*	−2.578 (−4.987, 1.088)

**Table 2:** The next generation matrix and the local reproduction number (*R*_*g*_) in each geographical area.

	High-latitude			Mid-latitude			Oceania		
	Mallard	Ducks	Others	Mallard	Ducks	Others	Mallard	Ducks	Others
Mallard	0.9877	0.0206	0.0153	0.9852	0.0206	0.0151	0.8076	0.0160	0.0122
Ducks	0.0759	0.7290	0.0492	0.0770	0.7299	0.0484	0.0631	0.5660	0.0389
Others	0.0810	0.0686	0.4657	0.0806	0.0692	0.4542	0.0618	0.0502	0.3615
*R*_*g*_	1.090			1.104			0.836		
